# Surveying Infections among Pregnant Women in the Niger Delta, Nigeria

**DOI:** 10.4103/0974-777X.68525

**Published:** 2010

**Authors:** FI Buseri, E Seiyaboh, ZA Jeremiah

**Affiliations:** *Haematology and Blood Transfusion Science Unit, Department of Medical Laboratory Sciences, Niger Delta University, Wilberforce Island, Bayelsa State, Nigeria*

**Keywords:** Bayelsa state, Co-infections, HBV, HCV, HIV, Pregnant women, Seroprevalence, South-South nigeria, Syphilis, Yenagoa

## Abstract

**Background:**

There is paucity of epidemiological data on infectious diseases among antenatal mothers in Bayelsa State of the Niger Delta, Nigeria.

**Aims:**

The aim of this study was to determine the seroprevalence of the serological markers Human immunodeficiency virus-antibody (HIV-Ab), Hepatitis B surface antigen(HBsAg), Hepatitis C virus antibody(HCV-A)and antibodies to T. pallidum among pregnant women in Yenagoa, Bayelsa State, South–South Nigeria.

**Settings and Design:**

This is a cross-sectional study which was carried out in Yenagoa city, the heart of the Niger Delta, Nigeria.

**Materials and Methods:**

Human immunodeficiency virus (HIV) antibodies were detected by using “Determine” HIV-1/2 test strip (Abbott Laboratories, Japan); hepatitis B surface antigen (HBsAg), antibodies to hepatitis C virus (anti-HCV) and antibodies to T. pallidum were carried out using ACON rapid test strips (ACON Laboratories, USA). All positive samples for HIV, HBV and HCV were confirmed using the Clinotech diagnostic enzyme-linked immunosorbent assay (ELISA) test kits (Clinotech Laboratories, USA), while all reactive samples to Treponema pallidum antibodies were confirmed by the Treponema pallidum hemagglutination (TPHA) test (Lorne Laboratories Ltd., UK). All test procedures were carried out according to the manufacturers’ instructions.

**Statistical Analysis Used:**

The data generated were coded, entered, validated and analyzed using Statistical Package for Social Science (SPSS), version 12.0, and Epi info. The seroprevalence of syphilis, HBsAg, HCV and HIV was expressed for the entire study group by age, sex and other demographic features using Pearson chi-square analysis. Values below 0.05 were considered statistically significant.

**Results:**

A total of 1,000 apparently healthy pregnant women aged between 15 and 44 years with a mean of 27.34±5.43 years were screened. In terms of percentage, 89.4% of the subjects were married, and 10.6% were without formal husbands. The overall seroprevalence of HIV, HBsAg, HCV and syphilis was found to be 4.1%, 5.3%, 0.5% and 5.0%, respectively.

**Conclusions:**

High prevalence of some infectious diseases was observed in the present study, which may pose serious health risk to women of reproductive age in this region. It is important to point out that there is need to improve antenatal care of pregnant women by mandatory screening for these infectious diseases.

## INTRODUCTION

Seroprevalence of HIV, HBV, HCV and syphilis infections is well recognized worldwide but has been reported to be more common in developing countries in Africa and Asia.[1–5] With the advent of highly active antiretroviral therapy (HAART), life expectancy of patients with HIV has increased, and the focus has now shifted to the management of concurrent illnesses such as chronic HBV and HCV infections and syphilis, which have the potential to increase long-term morbidity and mortality.[6–8] Moreover, epidemiologic studies have demonstrated that genital ulcers associated with primary syphilis are associated with an increased risk of HIV acquisition.[9]

Pregnant women are considered a sentinel population, because they are a relatively unselected population, for whom prevalence data may be extended to the general sexually active heterosexual population.[10] Failure to diagnose and treat these devastating disease agents at an early stage may result in serious complications and sequelae, including infertility, fetal wastage, ectopic pregnancy, anogenital cancer and premature death, as well as neonatal and infant infections.[11] Hence this cross-sectional study was undertaken with the aim of determining the seroprevalence of HIV, HBV, HCV and syphilis infections among women of childbearing age in Yenagoa, Bayelsa State, South–South Nigeria.

## MATERIALS AND METHODS

### Study population

A total of 1,000 apparently healthy pregnant women aged between 15 and 44 years (mean ± SD, 27.34±5.43 years) who were attending the antenatal care unit of the Family Support Programme (FSP) Clinic, Yenagoa, the capital city of Bayelsa State, for the first time during the current pregnancy were consecutively recruited for this study after obtaining their informed consent. Exclusion criteria included history of nonpregnancy, previous history of surgery and blood transfusion. Demographic data such as name, age, gravity, parity, gestational age, marital status, occupation and educational level were obtained via an interview-administered questionnaire.

### Sample collection

Three millimeters (3 mL) of venous blood was collected from each participant by clean venipuncture and dispensed into a clean, dry glass test tube and allowed to clot naturally at room temperature. The clotted blood samples were then spun in a centrifuge at 2,500 rpm for 5 minutes to separate the serum which was used for the analyses.

### Serological analyss

Screening for antibodies to human immunodeficiency virus (HIV) type 1 and 2 was carried out by using “Determine” HIV-1/2 test strip (Abbott Laboratories Ltd., Germany). Hepatitis B surface antigen (HBsAg), antibodies to hepatitis C virus (anti-HCV) and *Treponema pallidum* were assessed using ACON rapid test strips (ACON Laboratories, USA). All positive samples for HIV, HBV and HCV were confirmed using the Clinotech diagnostic enzyme-linked immunosorbent assay (ELISA) test kits (Clinotech Laboratories, USA), while all reactive samples to *Treponema pallidum* antibodies were confirmed by the *Treponema pallidum* hemagglutination (TPHA) test (Lorne Laboratories Ltd., UK). All test procedures were carried out according to the manufacturers’ instructions.

### Statistical analysis

The data generated were coded, entered, validated and analyzed using Statistical Package for Social Science (SPSS), version 12.0, and Epi info. The seroprevalence of syphilis, HBsAg, HCV and HIV was expressed for the entire study group by age, sex and other demographic features using Pearson chi-square analysis. Values below 0.05 were considered statistically significant.

## RESULTS

This study examined 1,200 blood samples from women of childbearing age, comprising 1,000 (83.3%) pregnant women who were attending the antenatal care unit of the Family Support Programme (FSP) Clinic, Yenagoa, between March 2008 and February 2009; and the remaining 200 (16.7%) were age-matched nonpregnant women residing in Yenagoa Metropolis, who served as controls. The age of the women ranged from 15 to 44 years with a mean of 27.34±5.43 and 29.94±6.88 years for pregnant and nonpregnant women, respectively [Table 1]. There was no statistically significant difference in their ages (*P*>0.05). The majority of women among the subjects and controls were in the 25-29 years age group (32.8% and 38.0%, respectively) [Table 1].

**Table 1:** Frequency distribution of subjects and controls according to age groups

Age group (years)	Subjects freq (%)	Controls freq (%)	
15–19	82 (8.2)	21 (10.5)	
20–24	221 (22.1)	34 (17.0)	
25–29	328 (32.8)	76 (38.0)	
30–34	275 (27.5)	37 (18.5)	
35–39	82 (8.2)	8 (4.0)	
40–44	12 (1.2)	24 (12.0)	
Total	1000 (100.0)	200 (100.0)	

**Population mean age (yrs)**	**Subjects**	**Controls**	***P* value**

	(*n*=1,ooo)	(*n*=200)	
	Mean ± SD	Mean ± SD	
	27.34±5.4	29.4±6.88	P>0.05

Table 2 shows the demographic characteristics of the pregnant and nonpregnant women studied. Majority of the study population were married (subjects, 89.4%; and controls, 73.0%), and over 70% of them possessed primary and secondary levels of formal education, with occupations as housewives, students, seamstresses, traders, civil and public servants. The subjects had the least percentage of women without formal education (3.7%), while students constituted the least occupational group. In relation to gravidity, more of the subjects were multiparous (66.8%), whereas more of the controls were primiparous (73.0%). All participants had already given birth and were therefore exposed to unprotected sexual intercourse.

**Table 2:** Demographic characteristics of the pregnant and nonpregnant women studied

Characteristics	Subjects (*n*=1,ooo)	Controls (*n*=200)	
**Biological age (Mean ± SD)**	27.34±5.4	29.4±6.88	P>o.05
**Gravida status**	**Number (%)**	**Number (%)**	
Primigravidae	332 (33.2)	146 (73.0)	
Multigravidae	668 (66.8)	54 (270)	
**Marital status**			
Married	894 (89.4)	146 (73.0)	
Single	106 (10.6)	54 (27.0)	
**Educational status**			
Primary	485 (48.5)	91 (45.5)	
Secondary	350 (35.0)	61 (30.5)	
Tertiary	128 (12.8)	30 (15.0)	
No formal education	37 (3.7)	18 (9.0)	
**Occupation**			
Housewife	501 (50.1)	105 (52.5)	
Trader	221 (22.1)	50 (25.0)	
Student	7 (0.7)	2 (1.0)	
Seamstress	100 (10.0)	23 (11.5)	
Civil servant	157 (15.7)	15 (7.5)	
Public servant	14 (1.4)	5 (2.5)	

Table 3 shows the sero-epidemiology of the infectious disease markers among the study population (pregnant and nonpregnant women). Two hundred four women, representing 17.0% of the study population, had one infectious disease marker or the other. The seroprevalence of HIV, HBV, HCV and syphilis infection was found to be 5.25%, 5.58%, 0.42% and 5.75%, respectively. Apart from HCV, the prevalence of HBsAg, HIV and syphilis were considered high. Anti-HCV was the least common of the infectious disease markers. All the 63 women who tested positive for HIV had only HIV type 1 infection. There was no HIV-2 or “HIV-1 and HIV-2” co-infection, indicating that HIV-1 was the predominant type in this environment.

**Table 3:** Prevalence of infectio us disease markers in the study population (*n*=1,200)

Infectious disease markers	Frequency	Percentage (%)
HBsAg	67	5.58
Anti-HCV	5	0.42
Anti-HIV	63	5.25
VDRL	69	5.75
Total	204	17.0

Comparison of the seroprevalence rates of the infectious disease markers between antenatal mothers (subjects) and nonpregnant control women shows higher seroprevalence rates among the controls than among the antenatal mothers (*P*<0.05) [Table 4]. Apart from HCV, the prevalence of HBsAg, HIV and syphilis was considered endemic among women of childbearing age.

**Table 4:** Comparison of the seroprevalence rates of the infectious disease markers between antenatal mothers (subjects) and nonpregnant control women

Percentage positivity				
Infectious agents	Subjects (*n*=1ooo)	Controls (*n*=200)	χ^2^	*P* value
HBV	50 (5.0%)	17(8.5%)	3.873	<0.05
HCV	5 (0.5%)	NIL (0%)	1.004	NA
HIV	41(41%)	22 (11.0%)	15.952	<0.0001
VDRL	53 (5.3%)	16 (8.0%)	2.242	<0.05

NA = Not applicable

Table 5 shows that every age group was afflicted with one infectious agent or the other, of which the 20-24 year age group was more affected (*P*<0.01). These findings further confirm the endemicity of these devastating disease agents in this locality and the predominance among younger age groups.

**Table 5:** Comparison of seroprevalence rates of the infectious disease agents in relation to age groups of pregnant women (*n*=1000) and nonpregnant women (*n*=200)

Age groups (years)	Infectious disease agents (Subjects)				Infectious disease agents (Controls)			
	HBsAg *n* (%)	HCV *n* (%)	HIV *n* (%) VDRL *n* (%)	VDRL *n* (%)	HBsAg *n* (%)	HCV *n* (%)	HIV *n* (%)	VDRL *n* (%)
15-19	4 (4.9)	0 (0.0)	6 (7.3)	6 (7.3)	2 (9.5)	Nil (0)	1 (4.8)	3 (14.3)
20-24	19 (8.6)	5 (2.3)	9 (4.1)	11 (5.0)	4 (11.8)	Nil (0)	6 (17.6)	1 (2.9)
25-29	16 (4.9)	0 (0.0)	9 (2.7)	17 (5.2)	9 (11.8)	Nil (0)	8 (10.5)	7 (9.2)
30-34	8 (2.9)	0 (0.0)	14 (5.1)	14 (5.1)	Nil (0)	Nil (0)	6 (16.2)	3 (8.1)
35-39	3 (3.7)	0 (0.0)	3 (3.7)	4 (4.9)	Nil (0)	Nil (0)	1 (12.5)	Nil (0)
40-44	0 (0.0)	0 (0.0)	0 (0.0)	1 (8.3)	2 (8.3)	Nil (0)	Nil (0)	2 (8.3)
Total	50 (5.0)	5 (0.5)	41 (4.1)	53 (5.3)	17 (8.5)	Nil (0)	22 (11.0)	16 (8.0)
Chi-Square	9.507	17.713	4.933	0.993	5.765	NA	6.400	3.161
*P* value	0.09ns	0.003**	0.424ns	0.963ns	0.330ns	NA	0.269ns	0.675ns

** ‐Significant at *P*<0.001; NS ‐ Not significant; NA ‐ Not applicable

In Table 6 the seropositivity rates of HCV and HIV among the primigravidae were higher than those among multigravidae (*P*<0.05); whereas HIV seropositivity was higher among the single mothers (7.5%) than among the married (3.7%) (*P*<0.05). As regards level of education, Table 6 shows a significant relationship between level of education and prevalence of infectious disease markers among pregnant women, especially with regard to HBsAg and Syphilis (*P*<0.0001 and *P*<0.05, respectively). Pregnant women with only primary level of education were more affected than women with other levels of education. In terms of occupation, housewives and students were more affected than other categories of occupations, especially with regard to HBsAg, HCV and HIV. Prevalence of VDRL was not statistically significant.

**Table 6:** Frequencies of the infectious disease agents in relation to demographic characteristics. Number and % age of seropositivity of infectious agents within age groups

Gravidae status	HBsAg *n* (%)	HCV *n* (%)	HIV *n* (%)	VDRL *n* (%)
Multigravidae (*n*=668)	33 (4.9)	1 (0.1)	21 (3.1)	38 (5.7)
Primigravidae (*n*=332)	17 (5.1)	4 (1.2)	20 (6.0)	15 (4.5)
Total = 1,000	50 (5.0)	5 (0.5)	41 (4.1)	53 (5.3)
Pearson Chi-Square	0.015	4.963	4.680	0.605
*P* value	0.902^ns^	0.02*	0.03*	0.437^ns^
**Marital status**			
Married (*n*=894)	45 (5.0)	5 (0.6)	33 (3.7)	50 (5.68)
Single (*n*=106)	5 (4.7)	0 (0.0)	8 (7.5)	3 (2.8)
Total = 1,000	50 (5.0)	5 (0.5)	41 (4.1)	53 (5.3)
Chi square value	0.020	0.596	3.583	1.441
*P* value	0.888^ns^	0.440^ns^	0.05*	0.230^ns^
**Education**			
Primary (*n*=485)	28 (5.8)	3 (0.6)	22 (4.5)	32 (6.6)
Secondary (*n*=350)	13 (3.7)	2 (0.6)	14 (4.0)	11 (3.1)
Tertiary (*n*=128)	2 (1.6)	0 (0.0)	3 (2.3)	5 (3.9)
No formal education (*n*=37)	7 (18.9)	0 (0.0)	2 (5.4)	5 (13.5)
Total = 1,000	50 (5.0)	5 (0.5)	41 (4.1)	53 (5.3)
**Occupation**			
Housewife (*n*=501)	27 (5.4)	1 (0.2)	18 (3.6)	28 (5.6)
Trader (*n*=221)	17 (7.7)	0 (0.0)	9 (4.1)	10 (4.5)
Student (*n*=7)	1 (14.3)	0 (0.0)	1 (14.3)	0 (0.0)
Seamstress (*n*=100)	1 (1.0)	4 (4.0)	6 (6.0)	1 (1.0)
Civil servant (*n*=157)	4 (2.5)	0 (0.0)	2 (1.3)	14 (8.9)
Public servant (*n*=14)	0 (0.0)	0 (0.0)	5 (35.7)	0 (0.0)
Total = 1,000	50 (5.0)	5 (0.5)	41 (4.1)	53 (5.3)
Chi-Square value	10.896	27.537	41.870	9.300
*P* value	0.053*	0.000***	0.000***	0.098^ns^

* ‐Significant at *P*<0.05;

NS ‐ Not significant

## DISCUSSION

In this study, 4.1% of the pregnant women were seropositive for HIV type 1 and none for HIV type 2 or “HIV-1 and HIV-2” dual infection. This suggests that HIV type 1 is more prevalent in this geographical area, corroborating the report that HIV type 1 is the predominant type worldwide. The seroprevalence rate may be considered high and is similar to the 3.96% found among the pregnant women who were attending the antenatal clinic of the Amassoma General Hospital, Amassoma, in southern Ijaw local government area of Bayelsa state, Nigeria.[12] It may also be considered similar to the 3.5% seroprevalence found amongst the antenatal women in the University of Port Harcourt Teaching Hospital, Port Harcourt, Nigeria.[13] The seroprevalence rate in this study is also similar to the Nigeria national overall average prevalence of 4.6% reported among antenatal women in 2008.[14] However, the prevalence rate in this study is higher than the 3.0% found in Jos, north-central Nigeria.[14,15]

By contrast, the prevalence rate in this study is lower than the 5.4% found in Abakaliki, southeastern Nigeria.[16] It is also lower than the 5.2% found amongst pregnant women in the Federal Medical Centre, Yola, northeastern Nigeria.[15] and the 8.6% reported among the antenatal attendees at Akwa, Anambra State, Nigeria.[17] The HIV seroprevalence rate observed in this study does not also agree with the 8.7% reported among antenatal attendees in Tanzania[18] and is also markedly at variance with the 30% prevalence rate found among Malawian pregnant women in Blantyre.[19] Also by contrast, Todd *et al*[20] found no case of HIV infection in the 4,452 pregnant Afghan women studied in Kabul, and Sahaf *et al*.,[1] also did not find any case of HIV infection in the 680 pregnant Iranian women studied in Malekan. The 2008 Nigeria National HIV Seroprevalence Sentinel Survey among antenatal clinic attendees reported that Bayelsa state had HIV seroprevalence rate of 7.2%.[14]

From the above, it can be seen that there are wide geographical variations in the seroprevalence rates of HIV infection amongst pregnant women within and outside Nigeria. The variations may be a reflection of the differences in sexual practices and behavior, awareness of HIV infection and testing, sociocultural practices and accessibility to healthcare.

In this study, Pearson chi-square analysis showed no statistically significant difference in the distribution of HIV infection among the various age groups (*P*>0.05). However, the HIV seropositivity was higher (19.2%) among the subjects aged between 15 and 34 years, whilst none was found within the 40-44 years age group. This suggests that women who are at the peak of their reproductive years are more prone to HIV infection. This may be associated with higher sexual activities within these age groups. In all epidemiological studies, younger age has always proved to be the most important factor. The age of acquiring the infection is the major determinant of the incidence and prevalence rates. Since HIV prevalence among young pregnant women (15-24 years) is used as a proxy for measuring rates of new infections in a population,[14] the findings in this study suggest an increasing new rate of infection. This is in agreement with the Nigeria national HIV prevalence trend among pregnant women, among whom the average prevalent rate in 2005 was 4.4%; and in 2008, 4.6%[14] The finding of slightly rising prevalence of HIV/AIDS is also supported by the reports of Joint United Nations programme on HIV/AIDS and World Health Organization (UNAIDS/WH).[21]

This study did not find any correlation in terms of formal education and HIV seropositivity (*P*>0.05). This suggests that possession of formal western education among women is not a main risk factor. This collaborates the finding of highest prevalence rate among students.[22] Even though the Nigerian students studied were knowledgeable about contraction routes for HIV, they were not deterred from engaging in unprotected sexual intercourse. Data on sexual behaviors indicate that risky behaviors are very common in Nigeria, while condom use remains low.[23] In couples, insistence on use of condom during sex may be a sign of lack of trust towards the partner, which may generate acrimony. Unprotected sexual intercourse is the major route of human procreation among couples; and unfaithfulness on the part of any of the partners by way of being involved in unprotected extramarital relationship constitutes a risk factor.

This study found a correlation in terms of occupation and HIV seropositivity (*P*<0.0001). HIV seropositivity was found among subjects with all the occupations in this study (full-time housewives, traders, students, seamstresses, civil and public servants). The women public servants constituted the bulk of HIV seropositivity cases (35.7%), followed by students (14.3%). The reason for this is not clear, but it may suggest involvement in unprotected promiscuous sexual relationships, since the women had not experienced blood transfusion and surgery.

In relation to marital status, HIV seropositivity was higher among the single pregnant women (women without formal husbands) (7.5%) as compared to the married women (5.3%) (*P*<0.05). This is in agreement with the report of Swai *et al*.[18] Other studies have found higher prevalence among married women or formerly married women. Such variations may imply that marital status per se is not a risk factor for HIV infection but an indicator to consider the sexual partner as a risk factor. The Centre for Disease Control and Prevention reported that at least 38% of women infected are through unprotected heterosexual contact with HIV-positive partner.[24] On the other hand, marital life itself becomes a risk factor for those women who get infected by their HIV-infected spouse. The potential of intramarital HIV transmission is very high, taking into account unprotected sexual activity in married or cohabiting couples and high-risk behavior of male spouses.

This study found a higher HIV seropositivity among primigravidae (6.0%) as compared to multigravidae (3.1%) (*P*<0.03) This may be due to early marriage associated with shorter period between premarital sexual debut and marriage associated with lower vulnerability among multigravidae.

The study also found a statistically significant difference between the pregnant women who were HIV seropositive (4.1%) and the control nonpregnant women (11.0%) (*P*<0.0001). This suggests high HIV seropositivity among women of reproductive age in Yenagoa and calls for expansion of the current voluntary counseling and testing (VCT) services and early access to antiretroviral (ARV) drugs. The difference in HIV seropositivity between pregnant and nonpregnant women may be an indication that only those pregnant women who had the financial muscle were able to access antenatal care.

The implications of HIV infection in pregnancy are serious. HIV-seropositive pregnant women are significantly more likely to have recurrent vulvovaginitis, perineal tear, postpartum hemorrhage, puerperal infection, birth asphyxia and increased perinatal mortality.[16] There is also a great risk of vertical transmission during parturition and breastfeeding.[25] Some women who learn of their HIV positive status during pregnancy may want to choose to terminate their pregnancy.[26] HIV may also adversely affect pregnancy outcome, leading to spontaneous abortion, premature delivery, intrauterine growth restrictions and low birth weight infants.[27] The interventions in preventing the aforementioned consequences can only be applied to a woman whose HIV status is known. Therefore, determining the HIV status of pregnant women is a key factor to the success of any prevention program. The findings in this study therefore support the opinion that pregnant women should be screened for HIV infection at the first antenatal clinic visit so that adequate management can be planned for them.

### HBsAg

This study found 5.0% HBsAg seropositivity prevalence rate among the pregnant women and 8.5% among the nonpregnant women. Pearson chi-square analysis showed statistically significant difference in the distribution of HBsAg seropositivity between the subjects and controls (*P*<0.05), among whom the nonpregnant women showed higher seropositivity. This may suggest that pregnancy per se is not a risk factor, and other risky behaviors may be responsible. This finding in this study is at variance with the 9.3% reported among pregnant women in Anambra state, southeastern Nigeria.[17] The finding is, however, similar to the 4.3% prevalence found in the University of Port Harcourt Teaching Hospital, Port Harcourt.[28] The seroprevalence rate in this study is higher than the 1.6% seroprevalence rate reported among women of reproductive age in Rome, Italy[29] and the 1.9% found among pregnant women in Bali, Indonesia[30] or the 2.1% found among women at delivery at the University of Benin Teaching Hospital, Benin City, Nigeria.[31]

In this study, the pregnant women with no formal education were found to possess the highest HBsAg seroprevalence rate (18.9%) compared to those women with primary (5.8%), secondary (3.7%) or tertiary (1.6%) level of education (*P*<0.001) The HBsAg seroprevalence rate was found to decrease with ascending levels of education. The reason for this is not clear, but it may suggest more improved level of hygiene with higher levels of education. This finding is in agreement with the finding by Ezegbudo and co-workers,[17] who reported that women with more education had less prevalence of HBV infection. However, Todd and colleagues[20] have reported that women whose husbands had finished university education were more likely to have HBsAg, while women whose husbands had no formal education were less likely to have HBsAg. This study did not screen the spouses of the seropositive women; probably such an assessment may be necessary to authenticate their claim. All occupational groups were affected except the public servants, and students had the highest seroprevalence (*P*<0.05); indicating a possibility of sharing common routes of infection, for example, sexual contact and clustered living conditions. Students were also found to have the highest seroprevalence rate in the study by Ezegbudo and co-workers[17] as compared to individuals with other occupations. Vertical transmission of HBV infection is thought to be a major mode of transmission in endemic areas.[32]

Among the subjects, HBsAg seroprevalence was found in all the age groups except those in the 40-44 years age group. The 20-24 years age group had the highest seroprevalence, viz., 8.6%; however, there was no statistically significant difference between individuals of various age groups (*P*> 0.05).

In relation to marital status, there was no statistically significant difference between the single and married pregnant women (*P*>0.05). This may suggest that pregnancy per se is not a risk factor. Since HBV is transmitted mainly through body fluids and these seropositive women had not been exposed to blood transfusion and surgery, unprotected heterosexual relationship with an infected partner or vertical transmission was a more culpable risk factor. There was also no statistically significant difference in the HBsAg seropositivity between primigravidae and multigravidae (*P*>0.05).

### HCV

With regard to HCV seroprevalence, this study found 0.5% seroprevalence among the subjects (pregnant women) and none (zero percent) among the control nonpregnant women. The finding suggests low prevalence of HCV infection among women of reproductive age in this geographical area. This prevalence rate is in agreement with the 0.31% seroprevalence found among intrapartum women in Kabul, Afghanistan.[20] However, the finding is not in agreement with the 2.4% seroprevalence found among women of reproductive age in Rome, Italy.[29] The finding in this study is even higher than the 0.04% found among 2,450 pregnant women in Bali, Indonesia.[30]

The 0.5% anti-HCV seroprevalence rate found among the pregnant women in this study is not in conformity with the 9.2% found among the pregnant women who were attending the Ladoke Akintola University of Technology Teaching Hospital, Osogbo, Osun State, a relatively new tertiary hospital in southwestern Nigeria.[33] The anti-HCV prevalence in this study is also lower than the 1%-2.6% earlier reported in similar studies on pregnant women from the Guinea and Cote d’Ivoire,[34,35] two countries in the same West African sub-region with Nigeria. This prevalence rate in this study is also lower than the range of 3.9%-13% reported in pregnant women from the non-West African countries of Tanzania, Egypt, Congo, Malawi and Cameroon.[36] These variations, noticed in different parts of Sub-Saharan Africa, may be related to the peculiarities in the modes of transmission of HCV dictated by sociocultural practices and environmental factors. These disparities may also be attributed to different epidemiologic methods of study; it should be pointed out that the choice of the serological algorithm to determine HCV seroprevalence is of great importance in developing countries, where inter-current infections contribute to false-positive enzyme immunoassay (EIA) results.[37]

The anti-HCV seroprevalence occurred only in 2.3% of the subjects in the 20-24 years age group, as shown in Table 4.19. The reason for its occurrence only in this age group is not clear, but this rate may not be far from the general low seroprevalence rate. Statistical analysis did not show any significant difference among the various levels of education (*P*>0.05). In relation to the various occupations of the subjects, seamstresses had the highest seroprevalence rate (4.0%), followed by housewives (0.2%) (*P*<0.001).

In relation to marital status, anti-HCV seroprevalence was found only among the married pregnant women (0.6%). This may be due to the general low seroprevalence rate of HCV infection in this geographical area, and it may also suggest that the role of sexual transmission in the spread of HCV seems to be limited. This is because single women are believed to be indulging in more sexual activities than the married women. The limited epidemiological evidence of HCV sexual transmission is consistent with the fact that HCV genetic sequences are seldom detected in semen or vaginal secretions of infected people.[38] On the other hand, there were more primigravidae (1.2%) that had anti-HCV seropositivity as compared to the multigravidae (0.1%) (*P*<0.02). The reason for this is not clear but may suggest individual variations in the susceptibility to the infection. Evaluation of any probable influence of HCV on the anemic status of the subjects did not show any statistically significant difference (*P*>0.05).

In the meantime, measures such as proper screening of blood and blood products for HCV, environmental sanitation and the discouragement of unnecessary and unsupervised parenteral injections and skin beautification with scarification marks should be promoted as means of reducing acquisition of HCV infection in the population.

### Syphilis

The overall prevalence of anti-syphilis sero-reactivity in this study was 69 out of 1,200, representing 5.8%, and is considered high. This may suggest the presence of risky sexual practices in the general population, because syphilis sero-reactivity is a “life style” indicator. The control nonpregnant women had higher seroprevalence rate (8.0%) than the pregnant women (5.3%), which difference was statistically significant (*P*<0.05). A possible explanation for the difference is population selection bias; women who visit antenatal clinics may pay more attention to their health and may have different socioeconomic characteristics than women who do not visit antenatal clinics. In addition, several risk factors that increase prevalence of sexually transmitted diseases, such as variable sexual contacts, low use of condoms and increased mobility of the population, are present in Yenagoa.

The syphilis sero-reactivity rate among the pregnant women in this study (5.3%) is higher than the 1.5% reported among antenatal attendees in Nicaragua[39] and the 4.3% found among the pregnant women in Francistown, Botswana.[40] The seroprevalence rate in this study, however, is lower than the 7.3% reported among antenatal clinic attendees in Tanzania.[18] The 5.3% seroprevalence rate found in this study is in agreement with the 5.0% reported among pregnant women in Blantyre, Malawi.[19] In Africa, the reported prevalence of syphilis in pregnancy ranges from 3.6% to 19% in antenatal clinics,[41] where congenital syphilis may account for about 1% of admissions to pediatric wards. Some epidemiologists have suggested that the increase in syphilis incidence may be a harbinger of an as yet unrecognized increase in HIV cases.

All age groups were affected, without any statistically significant differences (*P*>0.05) in this study, whereas Swai and colleagues[18] reported age-specific seroprevalence of syphilis, in which the individuals in the higher age group had higher seroprevalence. It seems that people start having unprotected sex at a young age and have multiple sexual relationships, even within marriage. Although the number of sexual partners a woman had was not determined, women alone cannot be singled out for blame. This is because the women could have been infected by their steady sexual partners. The number of partners and behavior of the woman’s partner could be more important than the number of partners of the woman herself. There is that male ego to keep multiple sexual partners or concubines in this locality and Nigeria in general.

Marital status, gravidity and occupation of the subjects did not appear to influence the prevalence of syphilis, and this finding is in accord with the finding by Swai *et al*.,[18] However, among the occupations, civil servants had the highest seroprevalence compared to those with other occupations. This research work showed that syphilis seroprevalence decreased with increasing levels of education, from 13.5% among women with no formal education to 6.6% among women with primary education to 3.1% among those with secondary education and 3.9% among women with tertiary education, indicating a statistically significant difference (*P*<0.016). This finding is in conformity with the report by Swai *et al*.,[18] In terms of percentage, 11.5% of the subjects with syphilis sero-reactivity had mild-to-moderate anemia, which is probable because syphilis infection is associated with insidious occult blood loss.

Syphilis has long been known to be an important risk factor for adverse pregnancy outcome. The consequences of untreated maternal infection include stillbirth, low birth weight (LBW), preterm live birth and also congenital infection in a proportion of surviving infants. Historically, one third of pregnancies are believed to result in second-trimester spontaneous abortion or perinatal death; one third, in a congenitally infected infant; and one third, in an uninfected infant. Data from developing countries confirm that maternal syphilis still remains an extremely important cause of perinatal morbidity and mortality. The impact of untreated syphilis in pregnancy at the population level may be considerable. In a prospective population-based study in Malawi and a retrospective cohort study in Tanzania, 21% of perinatal deaths, 26%-51% of stillbirths, 24% of preterm live births, 17% of all adverse pregnancy outcomes and 11% of neonatal deaths have been attributed to untreated high-titer [a rapid plasma reagin (RPR) test titer of ≥1:8 and a positive treponemal assay] maternal syphilis.[41]

## CONCLUSION

In conclusion, high prevalence of some infectious diseases was observed in the present study, which may pose serious health risk to women of reproductive age in this region. Considering that all infected women were asymptomatic and unaware of their infections until being revealed in this study, it is important to point out that there is need to improve antenatal care of pregnant women by mandatory screening for these infectious diseases.
